# Methods for Assessing MAGL Enzymatic Activity: An Extensive Review of Past and Emerging Approaches

**DOI:** 10.3390/ijms26199829

**Published:** 2025-10-09

**Authors:** Giulia Bononi, Eva Landucci, Miriana Di Stefano, Lisa Piazza, Simone Bertini, Marco Macchia, Carlotta Granchi

**Affiliations:** 1 Department of Pharmacy, University of Pisa, Via Bonanno, 6, 56126 Pisa, Italy; e.landucci1@student.unisi.it (E.L.); miriana.distefano@farm.unipi.it (M.D.S.); lisa.piazza@phd.unipi.it (L.P.); simone.bertini@unipi.it (S.B.); marco.macchia@unipi.it (M.M.); 2 Department of Life Sciences, University of Siena, Via Aldo Moro, 2, 53100 Siena, Italy

**Keywords:** monoacylglycerol lipase, MAGL, enzymatic assays, biochemical methods, enzymatic activity

## Abstract

Monoacylglycerol lipase (MAGL) is a key serine hydrolase involved in lipid metabolism, catalyzing the hydrolysis of monoacylglycerols into free fatty acids and glycerol. MAGL plays a central role in regulating endocannabinoid signaling and lipid homeostasis, processes often dysregulated in cancer and other pathological conditions. In recent years, MAGL has emerged as a promising therapeutic target, particularly in oncology, where its inhibition has shown potential to impair tumor growth, metastasis, and inflammation-driven processes. Alongside the development of selective MAGL inhibitors, several biochemical methods have been established to measure MAGL enzymatic activity, providing essential tools for target validation and inhibitor characterization. In this review, we provide a comprehensive and critical overview of the main approaches developed for MAGL activity evaluation, including radiometric, chromatographic, colorimetric, fluorescence-based, bioluminescence-based, and activity-based protein profiling (ABPP) assays. For each method, we discuss principles, advantages, and limitations. This review aims to support researchers in the selection of the most appropriate assay strategy for their experimental needs, ultimately fostering the rapid and accurate development of novel MAGL inhibitors with potential applications in cancer therapy and metabolic disease management.

## 1. Introduction

The endocannabinoid system (ECS) is a widespread signaling network playing a key role in various physiological processes throughout the body, including pain perception, mood regulation, immune responses, and energy homeostasis [1,2]. The ECS consists of cannabinoid receptors, endogenous ligands, and the associated enzymatic apparatus responsible for maintaining energy homeostasis and cognitive processes [2]. Cannabinoid receptors, CB1 and CB2, are mainly distinguished by the amino acid sequences and by their distributions in different tissues [3,4]. CB1, cloned in rats by Matsuda et al. in 1990 [5], is particularly expressed in the central nervous system (CNS) in regions responsible for mental and physiological processes such as the hippocampus, cerebral cortex, cerebellum, basal ganglia, hypothalamus, and amygdala [6,7]. CB2, cloned in 1993 from human promyelocytic leukemia cells of the HL-60 lineage [8], is characterized by a sequence with approximately 44% homology to the CB1 polypeptide chain and is mainly located in peripheral tissues, specifically in immune system cells [9]. The two main endogenous ligands of the ECS, anandamide (AEA) and 2-arachidonoylglycerol (2-AG), are lipid-derived signaling molecules, produced on-demand, that act as retrograde neurotransmitters. Synthesis and degradation of these endogenous ligands involve different enzymatic reactions. They are mainly synthesized on demand through the cleavage of phospholipidic membrane and released to act as pro-homeostatic agents [10,11]. Subsequently, both AEA and 2-AG are degraded by a carrier-mediated uptake followed by hydrolysis. AEA is primarily hydrolyzed by fatty acid amide hydrolase (FAAH), whereas the main enzyme responsible for the hydrolysis of 2-AG is monoacylglycerol lipase (MAGL), which catalyzes 2-AG hydrolysis into arachidonic acid (AA) and glycerol [12]. AA serves as a precursor for pro-inflammatory prostaglandins, thereby linking MAGL activity to inflammatory responses [13]. Since physiological levels of 2-AG are substantially higher than those of AEA, MAGL has emerged as a central regulatory enzyme in both endocannabinoid and eicosanoid signaling [12].

### 1.1. MAGL Structure and Catalytic Mechanism

**Figure 1:**
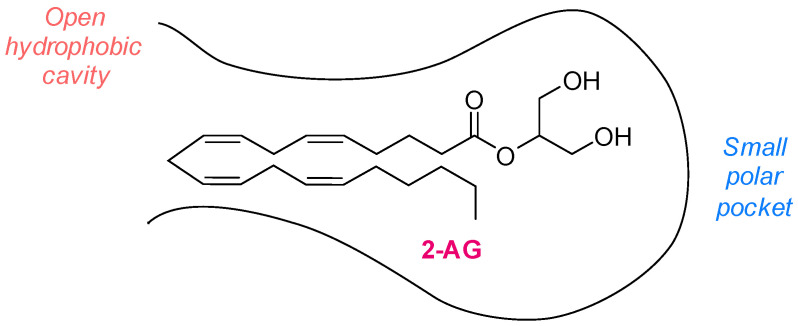
Two-dimensional representation of the MAGL active pocket.

MAGL is a membrane-associated enzyme part of the serine hydrolase superfamily, with its highest expression found in the brain, white adipose tissue, and liver in mice [14]. It was first purified from rat adipose tissue in 1976, representing a major milestone in its biochemical characterization [15]. Structural elucidation of mouse MAGL revealed that it consists of a polypeptide chain composed of 302 amino acids, with a molecular mass of 33.2 kDa. Later investigations demonstrated that the rat and human homologs are characterized by 303 amino acids, with a slightly increased molecular weight of 33.4 kDa. The degree of homology in terms of amino acid sequence between mouse, rat, and human isoforms is very high, with human and mouse MAGL being 84% identical and rat and mouse MAGL being 92% identical [16]. MAGL shows no homology with FAAH or other known amidases but shares the α/β-hydrolase fold, a tertiary fold that is typical of many lipases [17]. Specifically, MAGL is characterized by a catalytic triad of Ser122, Asp239, and His269 in its human isoform [18,19]. Within this active site, Ser122 acts as the nucleophile, facilitating substrate hydrolysis through interaction with their carbonyl groups. The enzyme’s secondary structure is predominantly defined by two key elements. The first is an α/β hydrolase core featuring a central β-sheet composed of seven parallel strands and one antiparallel strand, flanked by eight α-helices. Ser122 is located within the conserved Gly-X-Ser-X-Gly motif, commonly referred to as the ‘nucleophilic elbow’, a sequence critical for the precise positioning of the substrate [17]. The second key structural element is the lid domain, a flexible region composed of 75 amino acids that consists of two extended loop regions surrounding the active site. The lid domain regulates substrate access to the catalytic site, a role supported by crystallographic studies demonstrating that it adopts multiple conformations, reflecting its intrinsic flexibility. The lid also forms the entrance to a hydrophobic tunnel that leads directly to the catalytic Ser122, shaping a substrate-binding pocket specifically suited for accommodating 2-AG. The tunnel is predominantly hydrophobic, providing an optimal environment for accommodating the arachidonoyl chain of 2-AG, while its polar terminus interacts with the glycerol hydroxyl groups, a feature that plays a crucial role in substrate recognition, proper alignment within the catalytic pocket, and ultimately substrate specificity (Figure 1). Additionally, three cysteine residues (Cys201, Cys208, and Cys242) located near the catalytic triad play a key role in stabilizing the enzyme’s active conformation [12].

**Figure 2:**
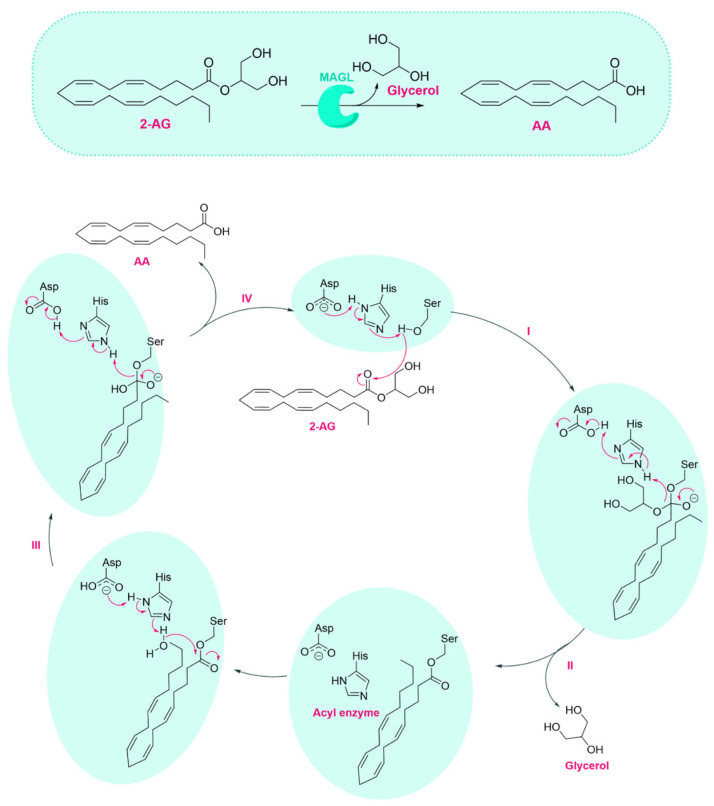
MAGL catalytic mechanism.

MAGL catalyzes the hydrolysis of 2-AG into AA and glycerol through a two-phase catalytic mechanism involving substrate binding and enzymatic hydrolysis [20]. Like other lipases, MAGL initiates catalysis through substrate interaction at the lipid-water interface, followed by the binding of a monomeric substrate molecule to its active site. As shown in Figure 2, once 2-AG is bound, its ester bond is cleaved via a nucleophilic attack by the hydroxyl group of Ser122, forming a tetrahedral intermediate stabilized by the enzyme’s oxyanion hole (Step I). This reaction is promoted by the catalytic triad (Ser122, His269, and Asp239) through a proton relay mechanism. Asp239 activates His269, which in turn deprotonates Ser122. Subsequently, as reported in Step II, the intermediate collapses, releasing the glycerol moiety and forming an acyl-enzyme intermediate, in which the arachidonoyl group remains covalently attached to the serine residue. Hydrolysis is completed by a nucleophilic attack by an activated water molecule on the acyl intermediate (Step III), liberating AA and regenerating the active enzyme (Step IV).

### 1.2. Therapeutic Potential of MAGL Inhibition

**Figure 3:**
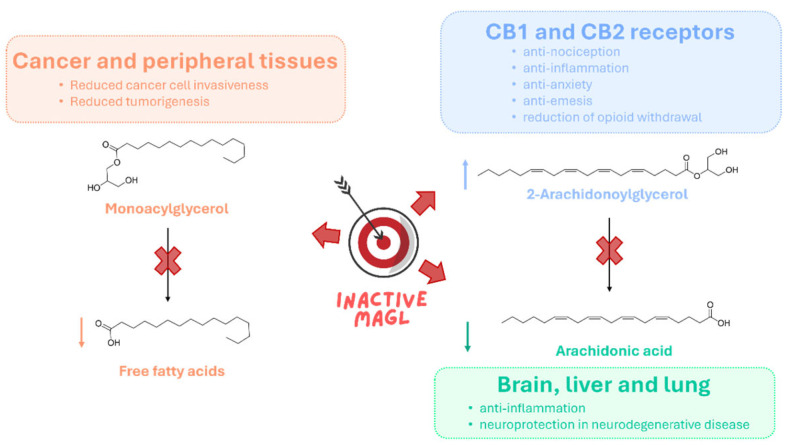
Schematic representation of the pathological functions of MAGL.

MAGL is a key enzymatic regulator of lipid signaling, with broad implications for several physiological and pathological processes, including neuroprotection, pain modulation, inflammation, tumor progression, and addiction [12,21,22]. Its primary function is the hydrolysis of 2-AG, a neuroprotective endocannabinoid, into AA, a precursor for the biosynthesis of pro-inflammatory prostaglandins. Pharmacological inhibition of MAGL elevates 2-AG levels while reducing AA availability, thereby enhancing cannabinoid receptor activation and suppressing pro-inflammatory prostaglandin synthesis. Through this dual control over endocannabinoid and eicosanoid pathways, MAGL inhibition has been associated with neuroprotective, anti-inflammatory, anti-nociceptive, and anxiolytic responses [23]. In preclinical models, MAGL inhibitors effectively reduce inflammatory pain [24] and show efficacy in multiple sclerosis and neuropathic pain [25]. In neurodegenerative diseases, MAGL inhibition exerts neuroprotective effects by mitigating neuroinflammation and preserving neuronal integrity. In Parkinson’s disease models, it prevents dopamine depletion and modulates glial-derived neurotrophic factor (GDNF) expression [26]. In Alzheimer’s disease, MAGL blockade reduces amyloid plaque burden and improves synaptic function, partly via microRNA-mediated mechanisms [27]. Similar neuroprotective benefits have been reported in models of multiple sclerosis [28], amyotrophic lateral sclerosis [29], and traumatic brain injury [30]. In cancer, MAGL is frequently overexpressed, particularly in aggressive and metabolically reprogrammed tumors. Elevated MAGL activity increases the availability of free fatty acids (FFAs), which serve as precursors for pro-tumorigenic lipids such as prostaglandin E2 (PGE2) and lysophosphatidic acid (LPA), supporting cancer cell proliferation, survival, and invasion [31]. High MAGL expression has been observed in melanoma [32], breast [31], ovarian [31], prostate [33], colorectal [34], hepatocellular [35,36], and nasopharyngeal carcinomas [37]. MAGL inhibition in these contexts impairs tumorigenesis and disrupts lipid-driven oncogenic signaling. Beyond its role in tumor progression, MAGL inhibition has shown potential in alleviating cancer-related symptoms such as pain and chemotherapy-induced nausea [38]. Collectively, these studies highlight MAGL as a key contributor to oncogenic processes and reinforce its potential as a therapeutic target. Recent studies have highlighted the involvement of MAGL in addiction, showing that its inhibition modulates endocannabinoid and dopaminergic signaling, thereby reducing drug-seeking behaviors and relapse vulnerability [39,40]. Together, these findings establish MAGL as a central metabolic regulator at the interface of endocannabinoid and lipid signaling (Figure 3). Its inhibition presents a versatile therapeutic strategy capable of restoring homeostatic signaling while counteracting pathological processes driven by dysregulated lipid metabolism.

### 1.3. MAGL Inhibitors

**Figure 4:**
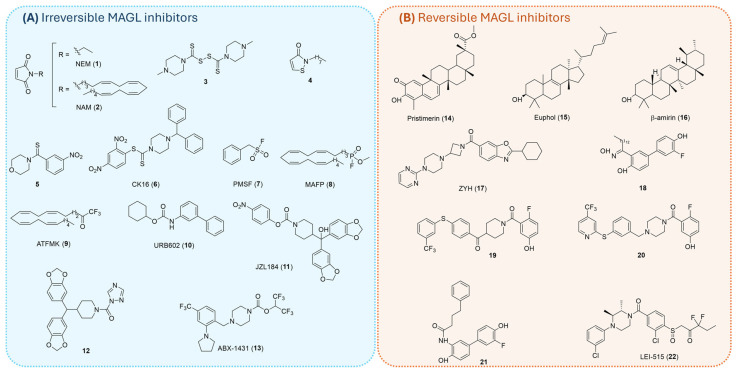
Structures of some representative irreversible (**A**) and reversible (**B**) MAGL inhibitors reported in the literature.

Given MAGL’s pivotal role in regulating 2-AG levels and the broad spectrum of physiological and pathological processes governed by this endocannabinoid, considerable efforts have been directed toward the development of potent and selective MAGL inhibitors. Consequently, numerous inhibitors with diverse chemical scaffolds have been identified, and they are generally classified based on their mode of inhibition as either irreversible (Figure 4A) or reversible (Figure 4B) inhibitors [22].

#### 1.3.1. Irreversible Inhibitors

A major class of irreversible MAGL inhibitors acts by covalently modifying nearby cysteine residues (Cys201, Cys208, and Cys242), thereby inhibiting the enzyme through stabilization of an inactive conformation or disruption of the active site’s structural integrity [19,41]. Among them, maleimide derivatives like *N*-ethylmaleimide (NEM, **1**, Figure 4A) and *N*-arachidonoylmaleimide (NAM, **2**, Figure 4A) demonstrated high potency by irreversibly modifying Cys242 via Michael addition to form an *S*-alkylated MAGL adduct [19,42]. Disulfide-based compounds, including disulfiram analog (**3**, Figure 4A), synthesized by Lambert and coworkers, inhibited MAGL through disulfide bond formation with cysteine residues (Cys208 and Cys242) [43,44]. King et al. identified isothiazolinone derivatives, exemplified by compound **4** (Figure 4A), showing a nanomolar inhibition potency against MAGL through a partially reversible inhibition mechanism. The authors proposed that compound **4** may form a reducible disulfide bond with one or more cysteine residues of MAGL [45]. Kapanda et al. synthesized arylthioamides exemplified by compound **5** (Figure 4A) [46] and 2,4-dinitroaryldithiocarbamates such as compound CK16 (**6**, Figure 4A) [47], both of them displaying strong selectivity for MAGL and an irreversible inhibition mechanism.

Another important class of irreversible MAGL inhibitors operates by targeting the catalytic serine residue (Ser122), exploiting its nucleophilic character to form covalent and stable adducts with electrophilic moieties within the inhibitor scaffold. Early examples include sulfonyl fluorides like phenylmethylsulfonyl fluoride (PMSF, **7**, Figure 4A), fluorophosphonates like methyl arachidonylfluorophosphonate (MAFP, **8**, Figure 4A), and trifluoromethylketones like arachidonyltrifluoromethylketone (ATFMK, **9**, Figure 4A) [17,48]. These compounds demonstrated high potency but suffered from broad reactivity, as they also inhibited numerous serine hydrolases, thereby limiting their selectivity and therapeutic potential. To overcome these limitations, medicinal chemists explored alternative electrophilic scaffolds with improved selectivity and pharmacokinetic profiles. During a screening of a library of *O*- and *N*-biphenyl carbamates, Piomelli and colleagues identified URB602 (**10**, Figure 4A) as a moderately potent inhibitor of 2-AG degradation [49]. A breakthrough came with the identification of JZL184 (**11**, Figure 4A) by Cravatt and colleagues, a carbamate derivative that exhibited high potency, excellent brain permeability, and remarkable selectivity over FAAH [50]. JZL184 (**11**) irreversibly carbamoylates Ser122, enabling sustained enzyme inhibition and providing a valuable tool for studying endocannabinoid signaling. Subsequent studies led to the development of urea-based inhibitors exemplified by compound **12** (Figure 4A), which can establish irreversible covalent bonds with the catalytic serine residue [51]. More recently, the development of ABX-1431 (**13**, Figure 4A) by Cisar et al. represents a significant advancement in the field. This compound acts as a potent and selective irreversible MAGL inhibitor, with favorable pharmacokinetic properties [52]. ABX-1431 is a lead compound selected for clinical evaluation due to its optimized activity and selectivity across multiple human protein targets. Phase I clinical trials have demonstrated that ABX-1431 is safe and well tolerated. It has now advanced into Phase II clinical studies targeting neurological indications such as Tourette syndrome, neuromyelitis optica, and multiple sclerosis [53].

#### 1.3.2. Reversible Inhibitors

The development of reversible MAGL inhibitors represents a significant advancement in the modulation of endocannabinoid signaling, offering improved safety profiles. Unlike irreversible inhibitors, which have been associated with CB1 receptor desensitization, pharmacological tolerance, and physical dependence due to prolonged elevation of 2-AG [54], reversible inhibitors allow for transient modulation of enzyme activity, thereby minimizing adverse central effects and helping to preserve overall ECS homeostasis. Various distinct classes of reversible inhibitors have been reported in the literature, each offering unique advantages in terms of potency, selectivity, and pharmacodynamic properties. Natural terpenoids were among the earliest identified. As shown in Figure 4B, Pristimerin and Euphol (compounds **14** and **15**, respectively), both isolated by Piomelli’s group, demonstrated significant MAGL inhibitory activity [55]. These compounds occupy a hydrophobic domain in the enzyme’s lid region and have inspired the exploration of related pentacyclic triterpenes, such as β-amyrin (**16**, Figure 4B) [56]. In addition to natural products, various synthetic small molecules have been developed as potent reversible inhibitors. Janssen Pharmaceutica introduced piperazinyl-azetidinyl amide ZYH (**17**, Figure 4B) as a promising reversible MAGL inhibitor [57]. This compound represents a key milestone in the structural investigation of MAGL, as it enabled the determination of a high-resolution co-crystal structure with human MAGL. This structural insight sheds light on the dynamic rearrangements of the enzyme, particularly within the catalytic site, upon ligand binding, providing valuable information for the rational design of new selective inhibitors [58]. In addition to ZYH, various synthetic small molecules have been developed as potent reversible inhibitors. Tuccinardi and colleagues explored innovative scaffolds for reversible MAGL inhibition, including salicylketoximes (**18**, Figure 4B) [59], benzoylpiperidines (**19**, Figure 4B) [60], benzylpiperazine (**20**, Figure 4B) [61], and *ortho*-hydroxyanilide derivatives (**21**, Figure 4B) [62]. Notably, benzylpiperazine-based compound **20** (Figure 4B) exhibited MAGL inhibitory activity in the low nanomolar range and a good selectivity over other ECS enzymes. Finally, Jiang et al. recently identified LEI-515 (**22**, Figure 4B), a novel peripherally restricted and reversible MAGL inhibitor developed through high-throughput screening (HTS) and structure-guided optimization. LEI-515 selectively elevates 2-AG levels in peripheral tissues without affecting the CNS, thus avoiding CB1-mediated adverse effects. In preclinical models, LEI-515 demonstrated potent anti-inflammatory and antinociceptive effects via CB2 receptor activation, without inducing physical dependence or tolerance [63].

## 2. Biochemical Methods to Assess MAGL Activity: From Conventional to Novel Approaches

Alongside the discovery of selective MAGL inhibitors, significant efforts have been devoted to developing robust biochemical assays for the precise evaluation of MAGL enzymatic activity [21]. These assessment methods play a crucial role in validating MAGL as a pharmacological target and are indispensable for profiling inhibitor potency and selectivity, thus becoming essential tools in the identification of novel and effective MAGL inhibitors.

The earliest approaches relied on radiolabeled substrates, such as 2-arachidonoyl-[^3^H]glycerol (2-[^3^H]AG) or 2-oleoyl-[^3^H]glycerol (2-[^3^H]OG), to monitor the hydrolytic release of radioactive glycerol or AA [64,65]. While highly sensitive, these assays posed significant challenges because of their reliance on radioactive materials, complex extraction steps, and incompatibility with HTS. Similarly, liquid chromatography techniques coupled with ultraviolet (LC/UV) [48,66] or mass spectrometry (LC/MS) [45,49,67,68] detection were introduced to quantify the enzymatic products of MAGL activity. Although these methods offered excellent specificity and were applicable to complex biological matrices, they were costly, time-consuming, and labor-intensive, rendering them impractical for large-scale inhibitor screening campaigns such as HTS.

To overcome these limitations, more accessible and rapid alternatives have been introduced, such as colorimetric [69,70] and fluorogenic [71,72,73,74,75,76,77,78] substrate-based assays, which enable real-time monitoring of MAGL enzymatic activity at lower operational costs. The probes employed in these assessment methods are designed to generate a detectable signal, such as absorbance or fluorescence, upon enzymatic cleavage, enabling continuous and rapid monitoring of MAGL activity and making these assays suitable for HTS. In particular, fluorogenic substrates have gained popularity due to their high sensitivity, low background signal, and ability to provide accurate kinetic parameters [79].

More recently, bioluminescence-based assays have emerged as cutting-edge tools for MAGL activity detection [80,81]. These techniques rely on synthetic substrates that produce a luminescent signal upon enzymatic hydrolysis, typically through luciferase-coupled reactions. Compared to traditional approaches, bioluminescent assays offer improved sensitivity, a broader dynamic range, and minimal background interference. Moreover, their ease of automation and scalability make them ideal for large compound library screening.

In addition to substrate-based assays, activity-based protein profiling (ABPP) has been introduced as a chemical biology approach to evaluate MAGL activity in complex proteomes and intact cells [82,83,84]. Unlike conventional biochemical methods, this cell-based methodology relies on covalent probes that specifically label the active site of MAGL, thereby allowing direct assessment of target engagement and inhibitor selectivity under physiologically relevant conditions.

Considering the significant acceleration in the identification and optimization of potent and selective MAGL inhibitors driven by technological advances in biochemical assessment methods, we provide a comprehensive and critical overview of the main techniques developed to date for the evaluation of MAGL activity. In this review, we focus on experimentally validated biochemical methods reported in peer-reviewed scientific articles, excluding data from patents. We discuss the underlying principles, advantages, and limitations of each approach, ranging from traditional methods to more recent assays.

This exhaustive analysis has the scope to guide researchers in selecting the most appropriate assay strategy according to their specific experimental needs, ultimately facilitating the discovery and development of increasingly potent and selective MAGL inhibitors.

**Figure 5:**
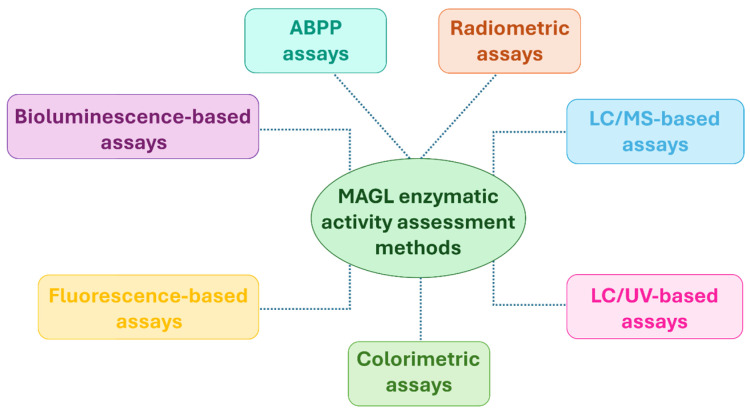
Biochemical methods for evaluating MAGL inhibitory activity.

In this section, we present and classify the biochemical assays available for evaluating MAGL activity according to the detection method employed: radiometric (Section 2.1), chromatographic (Section 2.2 and Section 2.3), colorimetric (Section 2.4), fluorometric (Section 2.5), and bioluminescent (Section 2.6) assays (Figure 5). Within each category, the methods are described in chronological order, from the earliest examples to the most recent advancements, thereby highlighting the progressive technical improvements and emerging trends in the field.

For completeness, Section 2.7 provides a concise overview of ABPP, outlining its underlying principles and highlighting its utility in evaluating MAGL activity and inhibitor selectivity (Figure 5).

**Figure 6:**
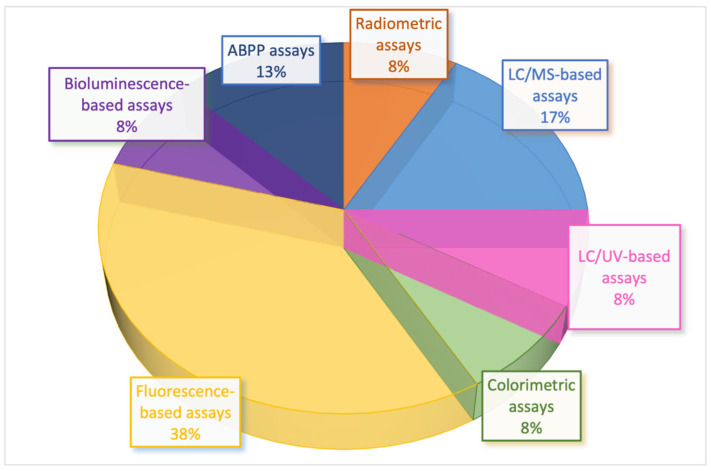
Distribution of biochemical methods used to assess MAGL inhibitory activity in the literature.

The analysis of the scientific literature reveals that approximately 38% of the published studies rely on fluorescence-based assays, while 17% employ LC/MS-based techniques and 13% are based on ABPP. The remaining 32% is almost equally distributed among radiometric, colorimetric, LC/UV-based, and bioluminescence-based methods (Figure 6; percentages refer only to the subset of methods analyzed and listed in Table 1). While a broad range of biochemical assays for MAGL have been developed in academic settings, only a subset has been widely implemented in pharmaceutical HTS campaigns. In practice, fluorescence-based assays, particularly those employing fluorogenic substrates or coupled enzyme reactions, remain the workhorse for large-scale primary screens due to their robustness, cost-effectiveness, and compatibility with miniaturized multi-well formats. In contrast, MS-based assays, although highly specific and capable of directly monitoring substrate/product conversion, are less frequently used as primary HTS methods because of lower throughput and higher operational costs. Instead, MS assays are often applied as orthogonal or secondary assays to confirm hits and reduce false positives. The preferential adoption of fluorescence-based methods highlights the balance between assay innovation and industrial feasibility, where sensitivity, reproducibility, and scalability are prioritized to meet the demands of pharmaceutical discovery programs.

### 2.1. Radiometric Assays

Radiometric assays were the earliest techniques developed to assess MAGL activity. Their high sensitivity makes them a reliable and accurate approach; however, the complexity of experimental procedures required, such as lipid extraction, fractionation by thin-layer chromatography (TLC), and the use of radiolabeled substrates, has limited their applicability for HTS.

The first radiometric method was described by Dinh and colleagues in 2002. In their protocol, tritium-radiolabeled substrates such as 2-[^3^H]AG or 2-[^3^H]OG were employed to quantify the radioactivity of the enzymatic products resulting from hydrolysis [64]. In detail, the supernatant protein was incubated in sodium phosphate buffer with 2-[^3^H]AG or 2-[^3^H]OG for 30 min at 37 °C. After incubation, the reaction was stopped, and the breakdown products were separated by organic solvent extraction using a chloroform/methanol (1:1, *v*/*v*) mixture. When 2-[^3^H]OG was used as a substrate, the released [^3^H]glycerol was measured in the aqueous phase by liquid scintillation counting (LSC) with quench correction. When 2-[^3^H]AG was employed, the organic phase was subjected to TLC separation, and radiolabeled products were analyzed by LSC. This radiometric assay was widely employed for determining MAGL inhibition activity and for identifying new MAGL inhibitors [43,85,86,87,88,89]; nevertheless, chloroform poses safety concerns and is incompatible with HTS formats based on plastic multiwell plates [65]. For these reasons, Brengdahl and co-workers explored a phenyl sepharose-based extraction procedure [65] as an alternative to the chloroform/methanol extraction previously proposed by Dinh et al. [64]. Phenyl sepharose is an adsorbent resin used in hydrophobic interaction chromatography (HIC) to separate molecules based on their hydrophobicity [90]. Its application in this context was inspired by a previously reported assay developed for acid sphingomyelinase, where it was used to separate a water-soluble product from a lipophilic substrate [91]. In their study, Brengdahl and colleagues demonstrated that the acidic phenyl sepharose extraction procedure enabled efficient separation of the lipophilic substrate 2-oleoylglycerol (2-OG) from the water-soluble product glycerol over a wide concentration range, providing a valid alternative to the previously reported chloroform/methanol extraction. The method proved robust and reproducible when applied to cytosolic fractions from rat brain, showing consistent pH sensitivity and inhibitor potency profiles. In particular, MAFP (**8**, Figure 4A) and ATFMK (compound **9**, Figure 4A), two irreversible MAGL inhibitors, were used to validate the developed protocol and were found to inhibit cytosolic 2-OG hydrolysis with pIC_50_ values of 8.68 and 5.79, respectively. In addition, the results obtained with ATFMK (**9**, Figure 4A) were consistent with those obtained using the standard chloroform/methanol extraction procedure conducted two years earlier by the same research group [85]. The primary limitations of this method include the suboptimal nature of phenyl sepharose suspensions for this application and the risk of false readings caused by accidental transfer of gel beads into the final aliquots; however, these issues can be mitigated by using filtration plates. The assay was further adapted to a 96-well format using C6 glioma cells, supporting its potential suitability for HTS of MAGL inhibitors targeting 2-OG hydrolysis. To avoid confounding results due to off-target hydrolytic activity from other enzymes such as FAAH, the authors recommended the use of cells overexpressing recombinant MAGL to confidently attribute the observed hydrolytic activity to this enzyme.

### 2.2. Mass Spectrometry (MS)-Based Assays

Recent advances in liquid chromatography as well as gas chromatography coupled with MS (LC/MS and GC/MS) have enabled the development of increasingly sophisticated and sensitive analytical methods for lipid quantification. These technologies have significantly improved the accuracy, sensitivity, and reliability of MAGL enzymatic activity assays, especially when using natural substrates such as 2-AG or 2-OG [92].

Among the earliest LC/MS-based assays, the method developed by Blankman and colleagues in 2007 represents a milestone in the functional characterization of 2-AG hydrolysis [67]. In this approach, whole-cell lysates were incubated with synthetic 2-AG in Tris buffer for 5 min at room temperature. The reaction was then quenched with chloroform/methanol (5:2, *v*/*v*), and the lipid products were extracted and analyzed using an Agilent 1100 series LC/MS system equipped with a reverse-phase C18 column with mass spectrometry detection in negative mode. The hydrolysis product AA was quantified by comparison to a pentadecanoic acid internal standard. Notably, the authors normalized MAGL activity to protein expression levels using average spectral count data from ABPP using the Multi-dimensional Protein Identification Technology (ABPP-MudPIT) proteomics [93] and SDS-PAGE band intensities, allowing accurate comparison between different serine hydrolases and correcting for expression variability across systems. This integrative strategy provided both functional and quantitative insights and enabled robust identification of MAGL as a major brain 2-AG hydrolase.

A few years later, alternative LC/MS-based assays were developed, focusing on simplified procedures while maintaining analytical robustness [45,49]. In the first method reported by King and co-workers in 2007 [49], MAGL activity was assessed using either purified *Escherichia coli* MAGL, HeLa-transfected cell lysates, or cerebellar membranes incubated with 2-OG as the substrate in Tris buffer. After a 10-min incubation at 37 °C, reactions were stopped with chloroform/methanol (2:1, *v*/*v*) containing heptadecanoic acid as an internal standard. Following centrifugation, the organic phase was dried under nitrogen, resuspended in chloroform/methanol (1:3, *v*/*v*), analyzed by LC/MS using a reverse-phase C18 column, and finally quantified by electrospray ionization (ESI) in negative ion mode. Although this assay did not include normalization for enzyme expression compared to that previously described by Blankman [67], it proved to be effective in detecting MAGL activity across different biological matrices.

A similar yet slightly modified approach was reported in a subsequent protocol by the same research group two years later [45]. This assay followed similar principles to the previous one but employed a smaller reverse-phase C18 column with a reduced flow rate and a higher column temperature. The internal standard was heptadecanoic acid, but in some experiments, heptadecenoic acid and 1-(3)-heptadecanoylglycerol were also used to improve quantification in complex mixtures.

A major step forward in chromatographic assays for MAGL activity was achieved by Kayacelebi and colleagues in 2017, who developed and cross-validated two highly sensitive stable-isotope dilution methods based on GC-MS and LC-MS/MS for quantifying the hydrolysis of 2-AG to AA [68]. These methods employed deuterium-labeled 2-AG (d_8_-2AG) as the MAGL substrate and measured the resulting d_8_-AA product, enabling specific detection of MAGL activity while avoiding interference from endogenous 2-AG and AA. In the GC-MS protocol, d_8_-AA was analyzed as its pentafluorobenzyl (PFB) ester using electron-capture negative-ion chemical ionization (ECNICI), whereas in the LC-MS/MS approach, the free acid form of d_8_-AA was directly monitored in selected reaction monitoring (SRM) mode. Unlabeled AA (d_0_-AA) was used as an internal standard in both MS-based assays. In consideration of the ubiquitous occurrence of AA in biological samples, the authors also tested the utility of the non-physiological eicosanoid 5,8,11,14-eicosatetraynoic acid (ETYA) as the internal standard for d_8_-2AG-derived d_8_-AA in tissue samples in the MAGL LC-MS/MS assay. The two developed methods were validated using recombinant MAGL, dog liver homogenates, and the human monocytic cell line Mono Mac 6.

This study demonstrated that using d_8_-2AG as the substrate and measuring d_8_-AA production through GC-MS and LC-MS/MS enables a reliable assessment of MAGL activity, with d_0_-AA serving as a robust internal standard. The authors confirmed that both methods, GC-MS (based on PFB-ester derivatization and ECNICI ionization) and LC-MS/MS (based on direct analysis of the free acid), provide comparable accuracy and precision in quantifying MAGL activity.

Due to the high abundance of free and esterified AA in biological samples such as liver tissue, ETYA was found to be a more suitable internal standard than d_0_-AA for tissue-based analyses, given its similar MS properties but different LC behavior. Notably, the study also showed that AA produced via MAGL-catalyzed hydrolysis of 2-AG could be efficiently converted to prostaglandin E_2_ (PGE_2_) by cyclooxygenase-2 (COX-2), supporting the concept of a MAGL/COX axis involved in inflammatory signaling in 2-AG-rich tissues like the brain.

Both GC-MS and LC-MS/MS assays showed strong performance, and both necessitated isolation from biological samples of d_0_-AA, d_8_-AA, and ETYA by solvent extraction. The GC-MS method required a time-consuming derivatization step (derivatization to their PFB esters with PFB-Br) but provided increased sensitivity thanks to ECNICI detection and improved selectivity after optimization of chromatographic separation. In contrast, LC-MS/MS allowed for faster analysis without derivatization, but it was more susceptible to matrix effects and required careful extraction and sample preparation to ensure accuracy. The authors concluded that both methods are reliable and complementary, with the choice depending on the biological context and available instrumentation.

### 2.3. Ultraviolet (UV)-Based Assays

Although MS offers high sensitivity and reliability, alternatives such as high-performance liquid chromatography (HPLC) coupled with UV detection have also been employed for evaluating MAGL activity [48,66].

One of the most relevant examples is the method developed by Saario and collaborators in 2004, which represents the first chromatographic approach using UV detection to assess the enzymatic hydrolysis of 2-AG in complex biological matrices [48]. In their protocol, enzyme assays were carried out at room temperature using cerebellar membrane protein incubated with 2-AG and other substrates (such as 1(3)-AG, AEA, noladin ether, or virodhamine). At 0 and 90 min, aliquots were collected, and enzymatic activity was quenched by adding acidified acetonitrile, which also prevented post-incubation acyl migration of 2-AG to its 1(3)-isomer. Following centrifugation, the supernatants were analyzed by HPLC using a C18 reversed-phase column and UV detection at 211 nm. The method showed good reproducibility and enabled clear differentiation among the hydrolytic profiles of various endocannabinoid substrates, supporting the presence of MAGL-like activity in cerebellar membrane preparations.

While this UV-based assay is less sensitive and specific than MS-based techniques, it offers a relatively simple and cost-effective option for studying MAGL activity and its inhibition, particularly when access to mass spectrometry instrumentation is not available. This UV-based assay has subsequently been employed in several studies, confirming its value for investigating MAGL activity in brain tissue and for the preliminary screening of potential inhibitors [41,94].

**Figure 7:**
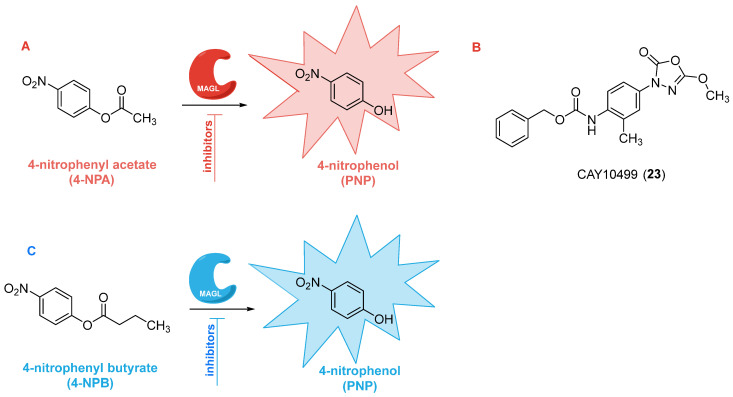
Colorimetric assays to assess MAGL inhibition activity by using 4-NPA (**A**) [69] or 4-NPB (**C**) [70] as chromogenic substrates. Structure of CAY10499 **23** (**B**) [69].

A significant advancement in UV-based chromatographic assays for MAGL activity evaluation was proposed by Del Carlo and co-workers in 2015 [66]. This method builds upon the classical colorimetric 4-nitrophenyl acetate (4-NPA) assay (see Section 2.4. Colorimetric assays and Figure 7A) [69], in which the enzyme-mediated hydrolysis of 4-NPA releases 4-nitrophenol (PNP, Figure 7A), a chromogenic product detectable by UV-Vis spectroscopy. Although this assay is simple, cost-effective, and compatible with 96- and 384-well plate formats commonly used for HTS, its sensitivity and specificity are limited by suboptimal detection wavelengths (typically greater than or equal to 405 nm) and spectral overlap with the substrate. Indeed, PNP displays a maximal absorbance at 315 nm, but 4-NPA also absorbs in the 270–320 nm range, complicating accurate quantification of the product in direct absorbance measurements. To address these limitations, the authors developed an HPLC/UV method that introduces a chromatographic separation before PNP detection, enabling selective and sensitive quantification of the hydrolytic product at its optimal absorbance maximum (λ = 315 nm), without interference from residual substrate or other matrix components. This implementation significantly increased the assay’s reliability, reproducibility, and accuracy. The method was rigorously validated against the radiometric assay using known MAGL inhibitors, revealing strong concordance between the IC_50_ values obtained with both techniques. Beyond quantification, the HPLC/UV assay also proved useful for characterizing inhibitor binding modes and distinguishing between reversible and irreversible inhibition, as well as competitive versus non-competitive mechanisms. Its versatility was further demonstrated under more physiologically relevant conditions: the authors employed the lipophilic ester 4-nitrophenyl decanoate (4-NPDo) as a MAGL substrate in the presence of Triton X-100 to mimic the interfacial environment of the enzyme at the membrane-cytosol boundary. Under these micellar conditions, substrate hydrolysis was enhanced, whereas inhibitor potency decreased, consistent with previous evidence that detergents can modulate enzyme-inhibitor interactions [72]. Although this method is not ideally suited for medium- or high-throughput screening, its main strength lies in its ability to avoid analytical interferences caused by the intrinsic UV absorbance of tested compounds. The chromatographic separation of substrate and product ensures that compounds such as the MAGL inhibitor pristimerin (**14**, Figure 4B) or other compounds such as flavonoids, curcumin, and polyphenols, which absorb significantly in the 300–450 nm range, do not bias PNP quantification. This is especially relevant given the growing interest in plant-derived MAGL inhibitors, which often contain chromophores that complicate direct UV-Vis analysis. In conclusion, the assay described by Del Carlo and colleagues represents a valuable and broadly applicable technique for early-stage pharmacological profiling of MAGL inhibitors.

### 2.4. Colorimetric Assays

Colorimetric assays have emerged as valuable tools for the rapid and cost-effective evaluation of MAGL activity and its pharmacological inhibition. Compared to classical approaches such as chromatographic or radiometric assays, which require specialized instrumentation and often involve radiolabeled substrates [64], colorimetric methods provide a high-throughput-compatible alternative, well suited for preliminary screening and kinetic characterization.

The first colorimetric assay was introduced in 2008 by Muccioli and colleagues, who employed 4-NPA as a chromogenic substrate [69]. In their comparative study, a series of 4-nitrophenyl alkyl esters with different chain lengths (C2–C12) were tested to identify the most suitable substrate. All compounds except 4-nitrophenyl laurate (C12) were hydrolyzed by MAGL, with 4-NPA displaying optimal solubility and reactivity. Hydrolysis of 4-NPA by MAGL releases *p*-nitrophenol (PNP), which can be quantitatively monitored by absorbance at 405 nm (Figure 7A). The substrate’s role was confirmed by its time-dependent hydrolysis, which followed a classic Michaelis–Menten kinetic profile, yielding *K*_m_ and *V*_max_ values of 0.20 mM and 52.2 μmol min^−1^ mg^−1^, respectively.

Optimized conditions (16 ng enzyme, 15 min incubation, 250 μM substrate) were established and successfully applied to test known MAGL inhibitors, such as MAFP (**8**, Figure 4A), NEM (**1**, Figure 4A), NAM (**2**, Figure 4A), and ATFMK (**9**, Figure 4A). The IC_50_ values obtained by using this method were consistent with previous results using purified human MAGL and radiolabeled 2-OG as the substrate. Notably, this assay enabled the identification of a novel inhibitor, CAY10499 (compound **23**, Figure 7B), which irreversibly blocked MAGL with an IC_50_ of 0.50 µM [69].

In 2022, Rodrigues and coworkers developed an alternative colorimetric assay based on 4-nitrophenyl butyrate (4-NPB) as a substrate (Figure 7C) [70]. The substrate 4-NPB is hydrolyzed by a broader spectrum of lipases, including MAGL, diacylglycerol lipase α (DAGLα), and α/β hydrolase 6 (ABHD6), thus providing an indirect yet reliable measure of overall 2-AG hydrolysis in brain tissue homogenates. In this assay, rat cerebral cortex membranes were resuspended in a HEPES-based buffer (20 mM HEPES, 1 mM CaCl_2_, pH 7.4) with a final protein concentration ranging from 0.4 to 2 mg/mL, and hydrolysis was monitored at 405 nm. The method showed a linear correlation between initial velocity (*V*_0_) and protein concentration (r^2^ = 0.74), confirming robustness across the tested range. Furthermore, the reaction was Ca^2+^-dependent, with activity reduced by 50% upon chelation with tetrasodium ethylene glycol-bis(β-aminoethyl ether)-*N*,*N*,*N*′,*N*′-tetraacetic acid (Na_4_EGTA), used as a selective calcium chelator, indicating involvement of calcium-regulated enzymes in 4-NPB hydrolysis. Importantly, known MAGL inhibitors, such as JZL184 (**11**, Figure 4A), effectively suppressed 4-NPB hydrolysis in cortical homogenates, further validating the assay’s applicability. Nevertheless, due to the promiscuity of 4-NPB hydrolysis across multiple lipase species, enzyme-specific contributions cannot be unequivocally assigned.

In summary, both 4-NPA- and 4-NPB-based colorimetric assays provide complementary strategies for studying MAGL activity and inhibition. The 4-NPA assay offers high specificity and detailed kinetic insights with purified enzymes, while the 4-NPB assay extends these applications to complex biological matrices. Together, these approaches represent robust, accessible, and versatile tools for pharmacological screening within the ECS.

### 2.5. Fluorescence-Based Assays

In recent years, fluorescence-based assays employing enzyme-responsive probes have emerged as essential tools in drug discovery, largely due to their compatibility with HTS platforms [79]. Their widespread adoption is driven by a combination of key advantages: outstanding sensitivity, rapid and real-time signal generation, minimal background interference, and adaptability to diverse assay formats [95,96]. Moreover, the broad availability of fluorophores with distinct excitation and emission profiles further enhances their utility. Collectively, these features enable non-invasive and continuous monitoring of enzymatic activity, establishing fluorescence-based methods as highly effective and flexible strategies for probing biochemical mechanisms.

Fluorescence-based assays can be classified according to their mode of signal acquisition, including total fluorescence intensity [97], fluorescence polarization [98], resonance energy transfer [99], fluorescence lifetime measurements [100], time-resolved fluorescence [101], or single-molecule techniques such as fluorescence correlation spectroscopy [102] and intensity distribution analysis [103]. Despite differences in detection strategies, the underlying principle remains the same: the enzymatic conversion of a non-fluorescent (or weakly fluorescent) substrate into a strongly fluorescent product, or vice versa. Fluorescent probes are typically composed of three functional domains: (1) a fluorophore, (2) a recognition element mimicking the enzyme substrate, and (3) a linker connecting the two. The recognition moiety must not only guide the probe to the active site but also ensure that the enzymatic reaction efficiently releases the fluorescent signal. The right balance between enzymatic affinity and release efficiency is critical to maximize probe sensitivity and selectivity while minimizing background fluorescence [79]. Although powerful, fluorescence-based assays are not without limitations. Signal interference arising from sample impurities or biomolecular autofluorescence can hide the specific signal of interest, underscoring the importance of rigorous sample preparation. Therefore, precise calibration, environmental control, and the use of photostable fluorophores are crucial for ensuring reproducibility and reliable data interpretation.

As previously discussed, historically, the quantification of MAGL activity relied on analytical methods such as radiometric assays using labeled substrates or chromatographic techniques like HPLC coupled to UV or MS detection. While these approaches are highly sensitive and accurate, they are often time-consuming, technically demanding, and require costly instrumentation or radioactive materials, making them poorly suited for HTS applications. To overcome these limitations, fluorescence-based assays for MAGL have been developed.

**Figure 8:**
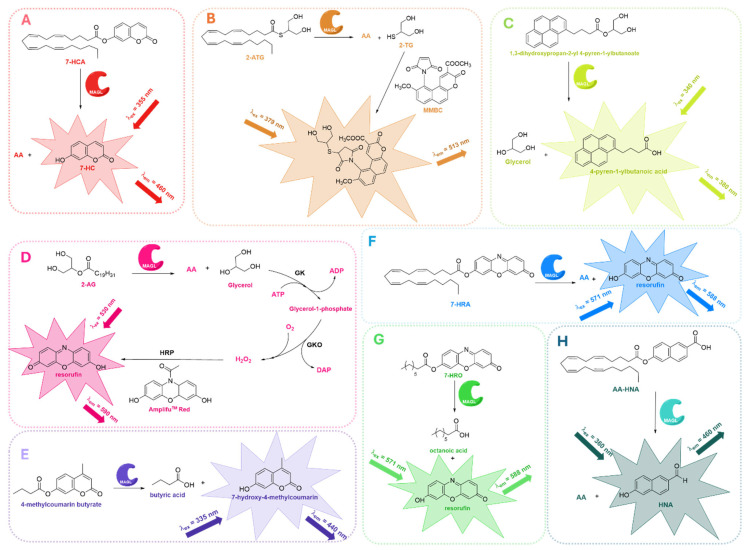
Fluorescence-based assays developed to evaluate MAGL inhibition employing 7-HCA (**A**) [71,74], 2-ATG (**B**) [73], 1,3-dihydroxypropan-2-yl 4-pyren-1-ylbutanoate (**C**) [72], 2-AG and GK (**D**) [106], 4-methylcoumarin butyrate (**E**) [75], 7-HRA (**F**) [76], 7-HRO (**G**) [77], or AA-HNA (**H**) [78] as fluorogenic substrates.

Early work employed coumarin-based probes. In 2008, Wang and colleagues introduced 7-hydroxycoumarinyl-arachidonate (7-HCA, Figure 8A), a fluorogenic analog of the endogenous substrate 2-AG, in which the glycerol backbone is replaced with a fluorescent coumarin moiety [71]. This modification allows MAGL to selectively hydrolyze the ester bond of 7-HCA, releasing AA and the highly fluorescent 7-hydroxycoumarin (7-HC, Figure 8A). Enzymatic activity can then be quantified by generating a calibration curve with known concentrations of 7-HC, enabling both reliable and precise measurements. The assay demonstrated a *K*_m_ of approximately 9.8 mM and a *V*_max_ of 1.7 mmol min^−1^ mg protein^−1^. MAGL hydrolysis of 7-HCA produces a strong signal, about 50 times higher than the baseline, with minimal variation (1% coefficient of variation). This combination of high sensitivity and reproducibility makes 7-HCA an excellent substrate for accurately studying MAGL activity, highly suitable for HTS applications.

Given the extensive utility of maleimides, known for their rapid and selective reactivity with thiol groups, some maleimide derivatives have long been applied in studies of protein structure and microenvironmental properties. Notably, the first maleimide-based fluorescent probes were introduced by Kanaoka and coworkers for protein structural studies [104], later finding use in microassays for glutathione *S*-transferase enzyme and in HPLC derivatization of thiol-containing compounds. Building on this foundation, in 2010 Casida and colleagues developed *S*-arachidonoyl-2-thioglycerol (2-ATG, Figure 8B), a thioester analog of the endocannabinoid 2-AG, designed for fluorometric assays [73]. This analog mimics 2-AG in both steric and electronic properties and acts as a suitable substrate for MAGL. Upon hydrolysis by MAGL, 2-ATG releases 2-thioglycerol (2-TG, Figure 8B), which can be quantitatively detected via fluorometric derivatization with methyl maleimido-benzochromenecarboxylate (MMBC, Figure 8B) [105]. This protocol has been applied in a dual approach, allowing the evaluation of both human recombinant MAGL and human brain membrane monoacylglycerol hydrolase activity. However, the dual-enzyme approach described by Casida et al. has seen limited use, leading researchers to prioritize single-enzyme assays.

In parallel with this approach, another important fluorescence-based assay for evaluating MAGL inhibition was introduced by Holtfrerich et al. [72]. In this protocol, a novel fluorogenic substrate was used, which is a structural analog of 2-AG in which the arachidonoyl group is replaced with a pyrene-labeled moiety (1,3-dihydroxypropan-2-yl 4-pyren-1-ylbutanoate, Figure 8C). Upon MAGL cleavage, this substrate releases 4-pyren-1-ylbutanoic acid, which can be easily detected. The assay was performed using human recombinant MAGL in the presence of Triton X-100, a surfactant that stabilizes the lipid-water interface, preventing any interference with enzymatic activity. Holtfrerich and colleagues demonstrated the utility of the method by validating it with well-characterized covalent MAGL inhibitors, including MAFP (**8**, Figure 4A), JZL184 (**11**, Figure 4A), and CAY10499 (**23**, Figure 7B). Thanks to its straightforward design, which does not require additional sample preparation, this assay represents a practical alternative to other fluorescence-based techniques.

In 2010, Savinainen and coworkers [74] refined and extended an earlier assay developed by Wang in 2008 (Figure 8A) [71]. By reanalyzing the original data, they observed that MAGL activity reached its maximum under alkaline conditions (pH 9.0–10.0), despite most assays having typically been performed at neutral pH (7.0–7.4). This observation is consistent with the fact that 7-HC is fully deprotonated and exhibits maximal fluorescence under alkaline conditions. To determine whether the increased MAGL activity at higher pH was simply due to enhanced fluorescence, the authors measured 7-HC fluorescence across a range of pH values from 7.4 to 9.0. As expected, fluorescence rose progressively with increasing pH. Furthermore, unlike conventional MAGL assays, the inclusion of bovine serum albumin (BSA) was found to be incompatible with the 7-HCA-based fluorescence method, as BSA itself amplified the fluorescent signal in a dose- and time-dependent manner, even in the absence of MAGL. The authors also highlighted that using a broad range of inhibitor concentrations, rather than a single high dose, allows for a more accurate and comprehensive assessment of inhibition reversibility.

It is important to mention the fluorescence-based assay for MAGL activity introduced by Navia-Paldanius and colleagues in 2012, who developed a sensitive fluorometric glycerol assay compatible with 96-well plate formats [106]. This method is based on detecting glycerol released from the hydrolysis of 2-AG, which is then converted into the fluorescent compound resorufin through a three-step enzymatic cascade. Specifically, glycerol is phosphorylated by glycerol kinase (GK) and oxidized by glycerol phosphate oxidase (GPO) to produce hydrogen peroxide, which finally reacts with Ampliflu™ Red in the presence of horseradish peroxidase (HRP) to generate resorufin (Figure 8D). The use of glycerol represents a key advantage of this assay, as natural substrates are generally preferred to ensure more accurate determination of inhibitor potency and enzyme kinetics, providing more reliable results compared to surrogate substrates. The assay exhibited excellent linearity for both glycerol concentration and protein amount, enabling real-time monitoring of MAGL activity in HEK293 cell lysates overexpressing human endocannabinoid hydrolases. Although the study primarily focused on ABHD6 and ABHD12, MAGL was included as a reference enzyme, and this work represents the first fluorescence-based MAGL approach using a natural substrate. Interestingly, the protocol developed by Navia-Paldanius et al. was recently used for the assessment of MAGL inhibition activity of the newly identified reversible MAGL inhibitor LEI-515 (**21**, Figure 4B) [63].

To expand the chemical space of fluorescent probes, Clemente et al. investigated various 4-methylcoumarin and coumarin-based acyl substrates, bearing aliphatic chains of different lengths, designed as mimics of 2-AG [75]. Among these, 4-methylcoumarin butyrate (Figure 8E) was selected as the most suitable candidate, since it displayed the best solubility profile and ensured reliable performance in kinetic assays. Hydrolysis of 4-methylcoumarin butyrate by MAGL releases butyric acid and 7-hydroxy-4-methylcoumarin, the latter showing absorbance peaks at 285 and 310 nm (ε_310nm_ of 6000 M^−1^ cm^−1^ in DMSO). A key innovation of this study was the use of orthogonal techniques, binding and activity assays, to rank MAGL ligands in parallel. Specifically, ThermoFluor, a label-free technique that monitors ligand binding through shifts in protein thermal stability, was combined with a fluorescent inhibition assay, thus providing complementary information on both target engagement and functional activity. This dual strategy reduces false positives and allows confident prioritization of MAGL inhibitors with therapeutic potential.

Three years later, Lauria and colleagues introduced a red-shifted fluorogenic substrate incorporating a resorufin fluorophore, which demonstrated high sensitivity and was well suited for MAGL activity screening (Figure 8F) [76]. The compound 7-hydroxyresorufinyl arachidonate (7-HRA, Figure 8F) proved to be a reliable substrate for fluorometric enzymatic assays, with the release of red fluorescent resorufin providing a high signal-to-noise ratio, reduced background interference, and excellent stability. Its suitability for HTS highlights its potential as a robust platform for inhibitor discovery. Furthermore, the red-shifted emission minimizes spectral overlap, offering a clear advantage over traditional blue-emitting probes.

To further refine the tools available for HTS, the same group later synthesized a library of resorufin-based esters containing acyl chains of different lengths [77], with the aim of identifying substrates with optimal enzymatic properties, good aqueous stability, and minimal spontaneous hydrolysis. Among the eleven newly developed candidates, 7-hydroxyresorufinyl octanoate (7-HRO, Figure 8G) emerged as the most promising one. It exhibited the highest enzymatic turnover, together with the most favorable kinetic parameters, making it particularly attractive for HTS applications. Docking simulations further supported its strong compatibility with the MAGL active site, indicating stable binding interactions. The probe was successfully validated using known MAGL inhibitors such as URB602 (**10**, Figure 4A) and MAFP (**8**, Figure 4A), confirming its practical applicability. This protocol proved to be a valuable tool for the identification of novel MAGL inhibitors in a recent study by Ottria et al., which led to the unexpected discovery of new lophine-based irreversible MAGL inhibitors [107].

In 2022, Deng and colleagues introduced a new fluorogenic probe, 6-hydroxy-2-naphthaldehyde-arachidonate (AA-HNA, Figure 8H), which incorporates 2-AG as a specific recognition moiety for MAGL into a fluorescent scaffold, 6-hydroxy-2-naphthaldehyde (HNA, Figure 8H) [78]. This innovative design enabled the development of a robust biochemical assay for the screening of potential MAGL inhibitors. The assay was applied to a library of 320 natural organic compounds, leading to the identification of new MAGL inhibitors with novel chemotypes. Furthermore, the authors applied ABPP as an orthogonal method to confirm the inhibitory activity against MAGL observed in the primary substrate-based screening. This validation step demonstrated the selective inhibition of MAGL over other enzymes involved in 2-AG and AEA hydrolysis, including ABHD6, ABHD12, and FAAH.

### 2.6. Bioluminescence-Based Assays

More recently, bioluminescence-based (BL) methods have attracted interest for their high sensitivity, low background signal, wide dynamic range, and relative simplicity of execution in HTS applications [80]. Firefly luciferases are commonly employed in these systems due to their ability to catalyze the ATP-dependent oxidation of *D*-luciferin (LH_2_), a compound containing a benzothiazole moiety, via a two-step enzymatic process [108]. In the first step, LH_2_ is adenylated by ATP to form luciferyl adenylate. In the second step, luciferyl adenylate is oxidized by molecular oxygen, leading to the formation of a dioxetanone intermediate that rapidly decomposes into CO_2_ and electronically excited singlet oxyluciferin. The excited oxyluciferin subsequently decays by photon emission, typically in the green to red region of the spectrum (yield 15–60%) [109,110]. This BL strategy has been widely adapted to various biological applications, including protein–protein interaction studies [111], gene expression monitoring [112], and in vivo imaging [113].

**Figure 9:**
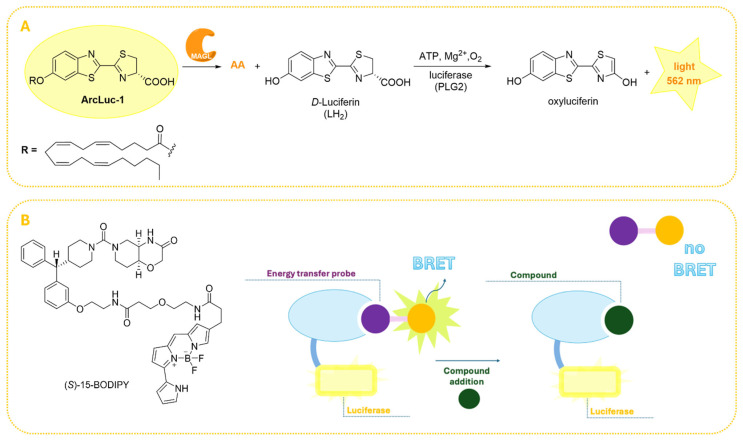
Bioluminescence-based strategies for assaying MAGL activity. (**A**) Schematic representation of the enzymatic hydrolysis of the synthetic substrate ArLuc-1 by MAGL, releasing luciferin (LH_2_), which is oxidized by PLG2 luciferase to produce bioluminescence [80]. (**B**) Principle of the NanoBRET assay and structure of (*S*)-15-BODIPY developed by Gazzi et al. [81].

Considering these advancements, Miceli and colleagues developed a novel BL assay to monitor MAGL activity both in vitro and in vivo (Figure 9A) [80]. This approach utilizes a synthetic luciferin derivative, arachidonoyl luciferin (ArLuc-1, Figure 9A), as a MAGL substrate. Upon enzymatic hydrolysis, free LH_2_ is released and immediately oxidized by the engineered luciferase PLG2, yielding a strong bioluminescent signal centered at 562 nm. PLG2 was selected for its thermal stability, pH resistance, and enhanced light output, features that make it particularly suitable for cellular applications. The performance of this assay was validated through kinetic studies using recombinant *h*MAGL, and the results fitted to the Michaelis-Menten model (*K*_m_ = 1.4 mM and *V*_max_ = 58 μmol min^−1^ mg^−1^) [80]. Additionally, the assay was validated with MAGL inhibitor JZL184 (**11**, Figure 4A) exhibiting an IC_50_ value of 260 nM, which is consistent with previously reported results by Lauria et al. [76] and Savinainen et al. [74], thereby confirming assay reliability.

Very recently, Gazzi and coworkers introduced a nanoluciferase-based bioluminescence resonance energy transfer (NanoBRET) platform that provides significant advantages for the pharmacological characterization of MAGL inhibitors in physiologically relevant contexts [81]. Unlike traditional assays employing purified enzymes, NanoBRET represents a powerful strategy allowing real-time assessment of inhibitor pharmacology in living cells. This assay provides critical insight into cellular dynamics such as influences of endogenous factors, compound permeability, non-specific binding, and intracellular metabolism, factors typically overlooked in cell-free assays [114]. As shown in Figure 9B, in the NanoBRET assay, target engagement is monitored by measuring energy transfer between a nanoluciferase (Nluc)-tagged protein [115] and a nearby small molecule tracer conjugated to a fluorophore. Competitive displacement of this tracer by an inhibitor results in a quantifiable decrease of energy transfer, thus enabling the determination of intracellular binding affinity. In this study, the researchers designed, synthesized, and validated a novel, cell-permeable, and reversible fluorescent probe specifically targeting MAGL [81]. The ideal probe was required to possess high cell permeability, selectivity for MAGL, good aqueous solubility, chemical stability, and well-characterized binding kinetics. The validation of this probe followed a reverse design strategy, starting from a scaffold with low nanomolar potency against both human and mouse MAGL isoforms and high passive cell permeability [116]. Structural insights from the X-ray crystal structure of *h*MAGL in complex with this scaffold revealed a terminal phenyl ring partially exposed to solvent, allowing for functionalization at the *meta* or *para* positions to introduce linkers or fluorophores. Based on these considerations, four compounds were synthesized, and (*S*)-15-BODIPY was selected as the probe with the most favorable binding kinetics, cell permeability, and high target engagement (Figure 9B). The NanoBRET assay was validated using a set of well-characterized MAGL inhibitors and subsequently applied to evaluate intracellular MAGL inhibition across a library of more than 1900 compounds. Comparative analyses with the well-established RapidFire MS biochemical assay [117] showed a strong correlation (R = 0.82), validating the assay’s predictive value for in vivo pharmacological relevance.

### 2.7. Activity-Based Protein Profiling (ABPP) Assays

**Figure 10:**
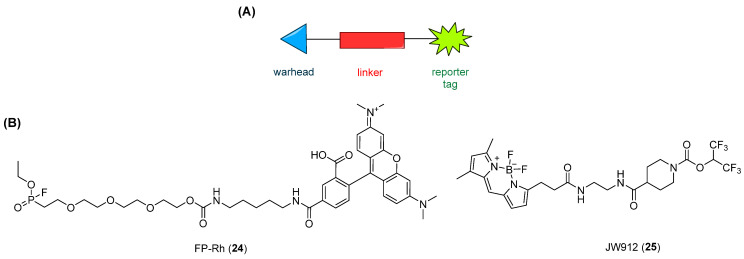
(**A**) General structure of an ABP: warhead (blue triangle), linker (red rectangle), and the reporter tag in green. (**B**) Structures of FP-Rh (**24**) and JW912 (**25**).

ABPP is a chemical proteomic approach that enables the assessment of enzyme activity in cells and tissues while simultaneously providing information on the selectivity of MAGL inhibitors across the entire proteome. Over the past decades, multiple applications of ABPP have been established, reflecting its interdisciplinary nature. This approach allows the simultaneous evaluation of inhibitors against numerous enzymes, thereby facilitating the determination of potency and selectivity in a time-efficient manner and ultimately accelerating the drug discovery process [84]. This methodology is based on the use of small-molecule probes that covalently bind to the active sites of enzyme families within complex proteomes. In contrast to the biochemical approaches described above, which require either natural or surrogate substrates to assess MAGL activity, ABPP is a cell-based strategy that employs activity-based probes (ABPs) able to bind to the active site of the target enzymes not only in cell lysates but also in intact cells and in animal tissues. This enables the evaluation of enzymatic activity and inhibitor selectivity without the need for a substrate [82,83]. ABPs (Figure 10) are typically composed of (1) a reactive “warhead” designed to recognize conserved structural motifs in enzyme active sites, such as electrophilic groups that target the catalytic nucleophilic serine of serine hydrolase family enzymes, and (2) a reporter tag, which enables target detection and characterization (e.g., biotin, fluorophore, or bioorthogonal moieties such as alkynes or azides suitable for modification through Click-chemistry reactions) [82]. Detection of labeled proteins is generally achieved using gel-based approaches, in particular when the ABP tag is a fluorescent one; LC-MS analysis when a biotin tag is employed; or imaging techniques when a fluorescent or radiolabeled tag is used [84]. Considering these features, ABPP has become a valuable alternative, as well as a complementary methodology to conventional substrate-based assays, to evaluate the activity and the selectivity of MAGL inhibitors both in vitro and in vivo [21].

Competitive ABPP is the most used ABPP strategy for the discovery and the optimization of MAGL inhibitors and generally includes gel-based and LC-MS-based approaches. In competitive ABPP, potential enzyme inhibitors are evaluated by their ability to compete with a covalent ABP for binding to the active site of the target enzyme [83].

At present, two structurally distinct ABPs have been developed to monitor MAGL activity in complex proteomes: fluorophosphonate (FP)-based probes, such as FP-rhodamine (FP-Rh, **24** Figure 10), and JW912 (**25**, Figure 10) [82,83,118]. FP-Rh (**24**, Figure 10) is a broad-spectrum serine hydrolase probe, and it can label different enzymes, including MAGL, whereas JW912 (**25**, Figure 10) is a fluorescent probe specifically designed from a MAGL inhibitor carbamate scaffold, exhibiting high selectivity for MAGL and ABHD6. Noteworthy, the application of ABPP technology allowed the identification of JZL184 (**11**, Figure 4A) [50], the first selective and in vivo-active MAGL irreversible inhibitor, and of ABX-1431 (**13**, Figure 4A) [52], the first-in-class carbamate-based irreversible MAGL inhibitor currently under investigation as an experimental therapeutic (see Section 1.3.1).

In summary, ABPP serves as a key strategy in MAGL inhibitor research, allowing for precise profiling of enzymatic activity and inhibitor selectivity in vitro and in vivo, thus accelerating the identification of compounds with therapeutic potential.

## 3. Concluding Remarks

MAGL is a key metabolic enzyme at the crossroads of endocannabinoid signaling and lipid homeostasis, with profound implications in cancer biology. Its upregulation in aggressive cancer cells and its pivotal role in sustaining pro-tumorigenic lipid networks underline the importance of this enzyme as a therapeutic target. Accordingly, MAGL inhibitors represent a novel and promising class of anticancer agents, potentially useful across different cancer types characterized by dysregulated lipid metabolism. Furthermore, MAGL plays a pivotal role in multiple pathologies by controlling the levels of the endocannabinoid 2-AG and downstream lipid mediators, thereby influencing neuroinflammation, pain signaling, and metabolic dysfunction. The continued development of potent and selective MAGL inhibitors has been consistently supported by the adoption of reliable and sensitive assay methods for MAGL enzymatic activity evaluation. In this regard, ABX-1431 (**13**, Figure 4A) provides a concrete example of how preclinical assays were instrumental in informing clinical development. The compound’s characterization as an irreversible MAGL inhibitor by ABPP experiments enabled the assessment of its potency and selectivity towards MAGL. The case of ABX-1431 highlights the translational value of ABPP: by confirming target engagement and guiding dose selection in preclinical models, these assays provided a solid foundation for subsequent clinical evaluation.

This extensive review of past and emerging methods for assessing MAGL enzymatic activity focuses on experimentally validated biochemical approaches reported in the literature, including radiometric, chromatographic, colorimetric, fluorescence-, and bioluminescence-based, and ABPP assays. For each approach, we discussed the fundamental principles, pros, and cons, which are summarized in Table 1. Particular attention should be paid to the choice of substrate, as surrogate substrates may not fully reflect the behavior of natural substrates, potentially influencing the accuracy of inhibitor potency measurements and enzymatic kinetics assessments.

In addition, Table 2 provides a summary of representative MAGL inhibitors (**1**–**22**, Figure 4), including their enzymatic inhibition values (IC_50_ or pIC_50_), type of inhibition (irreversible or reversible), the biochemical method applied, and the substrate used for activity assessment (natural or surrogate). This comparative overview is intended to provide the reader with a practical understanding of the approaches employed in the literature to evaluate MAGL inhibitors.

According to our analysis (Figure 6, Section 2, percentages refer only to the subset of methods analyzed and listed in Table 1), the most populated class of biochemical assays is fluorescence-based strategies, reflecting the growing interest in this approach, which offers high sensitivity, real-time monitoring, and compatibility with HTS, making it particularly attractive for large-scale inhibitor profiling. Recent advances in fluorogenic probes have further enhanced their utility in MAGL inhibitor research. For example, coumarin-based fluorogenic probes have emerged as preferred tools for real-time, non-invasive monitoring of target analytes in biological systems, owing to their optical characteristics, straightforward synthesis, efficient cell-membrane penetration, and favorable biocompatibility [119,120].

While fluorescence-based assays constitute the most extensively employed class of MAGL activity measurements, ABPP remains a powerful method for direct target engagement analysis across complex proteomes. In fact, cell-based ABPP approaches have received increasing attention due to their ability to assess enzymatic activity and inhibitor selectivity both in vitro and in vivo, offering substrate-independent and physiologically relevant insights. Despite these advantages, ABPP has some limitations, including the need for a suitable ABP, potential probe-related artifacts such as off-target labeling or incomplete proteome coverage, and lower throughput compared to simpler biochemical assays. It is therefore important to recognize these constraints when comparing ABPP to more established colorimetric and fluorescence-based assays. NanoBRET, as another emerging technique, represents a powerful strategy for real-time assessment of inhibitor pharmacology in living cells. This assay provides critical insights into cellular dynamics; however, NanoBRET also has its limitations, such as requiring specialized reagents (luciferase substrates) and higher costs compared to colorimetric or fluorescence-based assays. Therefore, ABPP and NanoBRET retain distinctive advantages as well as specific limitations, and their complementary strengths support the continued relevance of both techniques in the evolving methodological landscape. Continued methodological improvements will likely enhance the applicability and reliability of both ABPP and NanoBRET for studying MAGL and other enzymatic targets.

Looking ahead, there are several emerging strategies that could significantly enhance the toolkit for MAGL enzymatic assays, complementing the existing classical, fluorescent, bioluminescent, chromatographic, and ABPP methods. An interesting strategy is the structural biology-guided design of fluorescent probes. Recent works demonstrate the value of combining high-resolution structural information with probe design to obtain fluorescent or click-chemistry reporter units with high potency and selectivity. For example, using structure-based and reverse-design approaches, Hentsch et al. created a modular scaffold that binds MAGL with sub-nanomolar potency and tolerates attachment of different fluorophores without losing affinity or selectivity [121]. The same research group developed miniaturized fluorescent probes targeting MAGL by embedding a boron-dipyrromethene (BODIPY) moiety directly in the inhibitor scaffold; these probes show favorable drug-like properties (permeability and solubility), high cell selectivity, and picomolar potency [122]. These recent studies suggest that future probe development could increasingly leverage structural insights to optimize not only binding affinity but also kinetics, selectivity, cellular uptake, and potentially in vivo imaging performance. Although promising fluorescent probes for MAGL have been reported, there is still no translation into genetically encoded sensors for live-cell imaging. These sensors, which are reporter proteins that emit a measurable optical signal in response to enzyme activity, would require not only a domain responsive to MAGL substrates or products but also a strong signal, suitable kinetics, correct localization, and minimal interference. Likewise, microfluidic platforms, consisting of miniaturized systems that manipulate tiny fluid volumes in channels or droplets, could bridge the gap between simple in vitro assays and experiments in cells or tissues, improving throughput, reagent use, and experimental control.

Although biochemical and cellular assays represent the cornerstone for characterizing MAGL inhibitors, their translational value has intrinsic limitations. In vivo assays, while critical for assessing pharmacodynamic responses and behavioral outcomes, are often constrained by issues of variability, species differences, and the challenge of capturing long-term efficacy and safety. Moreover, a direct correlation between biochemical inhibition and therapeutic benefit is not always linear, as compensatory mechanisms within the ECS can attenuate or modify drug effects. These considerations highlight the need for integrated assay strategies that combine biochemical, cellular, and in vivo approaches to better predict clinical outcomes. Future developments in translational biomarkers may help bridge this gap and improve the predictive power of preclinical studies.

By critically examining the strengths and limitations of each technique, this review is intended to provide a practical resource to support decision-making in experimental design and to foster innovation in the development of next-generation MAGL inhibitors as promising therapeutic agents.

**Table 1:** Summary of biochemical methods used for the assessment of MAGL enzymatic activity: principles, advantages, and limitations.

Assessment Method	Substrate	Pros	Cons	Reference
*Radiometric assays*	Natural (2-AG or 2-OG) substrates	Very high sensitivity	- Complex experimental procedures (e.g., lipid extraction, fractioning by TLC, and radiolabeled substrates) - Costly - No real-time monitoring - Incompatibility with HTS	[64,65]
*Chromatographic (UV- or MS-based) assays*	Natural (2-AG or 2-OG) substrates	- High sensitivity and accuracy - Specific quantification of products	- Complex experimental procedures (e.g., lipid extraction and phase separation) - Costly - Low throughput - Not ideal for HTS - No real-time monitoring	[45,48,49,66,67,68]
*Colorimetric assays*	Surrogate substrates (4-NPA or 4-NPB)	- Cost-effective - Easy detection method (absorbance) - Simple setup - Real-time monitoring - HTS-compatible - No lipid extraction required	- Low sensitivity - Reduced accuracy due to the use of surrogate substrates	[69,70]
*Fluorescence-based assays*	Surrogate (7-HRA, 7-HRO, 4-methylcoumarin butyrate) and natural (glycerol) substrates	- Cost-effective - Easy detection method (fluorescence) - Real-time monitoring - HTS-compatible - No lipid extraction required - Possibility of using natural substrates under more physiological conditions	- Some compounds may interfere with fluorescence signal - Require optimized conditions - Less straightforward than colorimetric assays	[71,72,73,74,75,76,77,78,106]
*Bioluminescence-based assays*	Surrogate substrates (luciferase-coupled)	- Very high sensitivity - Broad dynamic range - Minimal background noise - Real-time monitoring - HTS-compatible - Amenable to automation	- Require specialized reagents (luciferase substrates) - Higher cost compared to colorimetric/fluorescent assays	[80,81]
*ABPP assays*	No requirement for substrates	- Proteome-wide selectivity profiling - Simultaneous assessment of enzymatic activity and inhibitor selectivity in a single experiment - Applicable to both in vitro and in vivo studies	- Availability of a suitable ABP - Potential probe-related artifacts (off-target labeling or incomplete proteome coverage) - Lower throughput compared to simple biochemical assays (gel-based ABPP assays)	[82,83,118]

**Table 2:** Key properties of representative MAGL inhibitors (**1**–**22**, Figure 4), including inhibition values, inhibition type, assay method, and substrate.

MAGL Inhibitor	Inhibition Activity	Inhibition Type	Assay Method	Detection Substrate	Reference
NEM (**1**)	IC_50_ = 53 µM	Irreversible	Chromatographic UV-based assay	Natural substrate: 2-AG	[41]
NAM (**2**)	IC_50_ = 0.14 µM	Irreversible	Chromatographic UV-based assay	Natural substrate: 2-AG	[41]
**3**	pIC_50_ = 6.96	Irreversible	Radiometric assay	Natural substrate: 2-OG	[44]
**4**	IC_50_ = 88 nM	Irreversible	Chromatographic MS-based assay	Natural substrate: 2-OG	[45]
**5**	pIC_50_ = 5.02	Irreversible	Radiometric assay	Natural substrate: 2-OG	[46]
CK16 (**6**)	pIC_50_ = 6.45	Irreversible	Radiometric assay	Natural substrate: 2-OG	[47]
PMSF (**7**)	IC_50_ = 155 µM	Irreversible	Chromatographic UV-based assay	Natural substrate: 2-AG	[48]
MAFP (**8**)	IC_50_ = 2.2 nM	Irreversible	Chromatographic UV-based assay	Natural substrate: 2-AG	[48]
ATFMK (**9**)	IC_50_ = 66 µM	Irreversible	Chromatographic UV-based assay	Natural substrate: 2-AG	[48]
URB602 (**10**)	IC_50_ = 223 µM	Irreversible	Chromatographic MS-based assay	Natural substrate: 2-OG	[49]
JZL184 (**11**)	IC_50_ = 8 nM	Irreversible	ABPP assay	No requirement for substrate	[50]
**12**	IC_50_ = 363 pM	Irreversible	Fluorescence-based assay	Natural substrate: 2-AG and GK	[51]
ABX-1341 (**13**)	IC_50_ = 0.014 µM	Irreversible	ABPP assay	No requirement for substrate	[52]
Pristimerin (**14**)	IC_50_ = 93 nM	Reversible	Chromatographic MS-based assay	Natural substrate: 2-OG	[55]
Euphol (**15**)	IC_50_ = 315 nM	Reversible	Chromatographic MS-based assay	Natural substrate: 2-OG	[55]
β-Amirin (**16**)	IC_50_ = 2800 nM	Reversible	Chromatographic MS-based assay	Natural substrate: 2-OG	[56]
ZYH (**17**)	IC_50_ = 0.010 µM	Reversible	Fluorescence-based assay	Surrogate substrate: 4-methylcoumarin butyrate	[57]
**18**	IC_50_ = 0.68 µM	Reversible	Colorimetric assay	Surrogate substrate: 4-NPA	[59]
**19**	IC_50_ = 1.26 nM	Reversible	Colorimetric assay	Surrogate substrate: 4-NPA	[60]
**20**	IC_50_ = 5.2 nM	Reversible	Colorimetric assay	Surrogate substrate: 4-NPA	[61]
**21**	IC_50_ = 0.34 µM	Reversible	Colorimetric assay	Surrogate substrate: 4-NPA	[62]
LEI-515 (**22**)	IC_50_ = 25 nM	Reversible	ABPP assay	No requirement for substrate	[63]

## Data Availability

No new data were created or analyzed in this study. Data sharing is not applicable to this article.
